# Treating Depression in Adolescents and Young Adults Using Remote Intensive Outpatient Programs: Quality Improvement Assessment

**DOI:** 10.2196/44756

**Published:** 2023-04-11

**Authors:** Michelle Evans-Chase, Phyllis Solomon, Bethany Peralta, Rachel Kornmann, Caroline Fenkel

**Affiliations:** 1 School of Social Policy & Practice University of Pennsylvania Philadelphia, PA United States; 2 Charlie Health, Inc Bozeman, MT United States; 3 Department of Behavioral Health Rutgers University New Brunswick, NJ United States

**Keywords:** depression, IOP, LGBTQ+, mental health, telehealth, youth, young adult

## Abstract

**Background:**

Youth and young adults face barriers to mental health care, including a shortage of programs that accept youth and a lack of developmentally sensitive programming among those that do. This shortage, along with the associated geographically limited options, has contributed to the health disparities experienced by youth in general and by those with higher acuity mental health needs in particular. Although intensive outpatient programs can be an effective option for youth with more complex mental health needs, place-based intensive outpatient programming locations are still limited to clients who have the ability to travel to the clinical setting several days per week.

**Objective:**

The objective of the analysis reported here was to assess changes in depression between intake and discharge among youth and young adults diagnosed with depression attending remote intensive outpatient programming treatment. Analysis of outcomes and the application of findings to programmatic decisions are regular parts of ongoing quality improvement efforts of the program whose results are reported here.

**Methods:**

Outcomes data are collected for all clients at intake and discharge. The Patient Health Questionnaire (PHQ) adapted for adolescents is used to measure depression, with changes between intake and discharge regularly assessed for quality improvement purposes using repeated measures *t* tests. Changes in clinical symptoms are assessed using McNamar chi-square analyses. One-way ANOVA is used to test for differences among age, gender, and sexual orientation groups. For this analysis, 1062 cases were selected using criteria that included a diagnosis of depression and a minimum of 18 hours of treatment over a minimum of 2 weeks of care.

**Results:**

Clients ranged in age from 11 to 25 years, with an average of 16 years. Almost one-quarter (23%) identified as nongender binary and 60% identified as members of the lesbian, gay, bisexual, transgender, queer (LGBTQ+) community. Significant decreases (mean difference –6.06) were seen in depression between intake and discharge (*t*_967_=–24.68; *P*<.001), with the symptoms of a significant number of clients (*P*<.001) crossing below the clinical cutoff for major depressive disorder between intake and discharge (388/732, 53%). No significant differences were found across subgroups defined by age (*F*_2,958_=0.47; *P*=.63), gender identity (*F*_7,886_=1.20; *P*=.30), or sexual orientation (*F*_7,872_=0.47; *P*=.86).

**Conclusions:**

Findings support the use of remote intensive outpatient programming to treat depression among youth and young adults, suggesting that it may be a modality that is an effective alternative to place-based mental health treatment. Additionally, findings suggest that the remote intensive outpatient program model may be an effective treatment approach for youth from marginalized groups defined by gender and sexual orientation. This is important given that youth from these groups tend to have poorer outcomes and greater barriers to treatment compared to cisgender, heterosexual youth.

## Introduction

Approximately 3 million youths were diagnosed with major depressive disorder (MDD) in 2019 [1], of whom 18% received treatment in that same year [2]. While micro-level factors undoubtedly contribute to low treatment rates at the individual level, treatment availability presents a structural barrier to all youth, particularly for those with crisis level or complex mental health needs. Such barriers include a lack of access to hospital-based treatment, with only 39% of hospital-based facilities nationwide serving youth younger than 18 years [3] and excessive wait times in youth-serving facilities averaging 6-8 weeks. A third barrier involves access to developmentally appropriate treatment, with only 36% of mental health programs designed specifically for children and adolescents, and only 22% designed for transition-age young adults [4]. Youth and young adults living in rural counties face even greater barriers, with only 1% of hospital-based facilities serving youth younger than 18 years located in rural areas [3]. These barriers reflect a worldwide neglect of adolescent mental health and contribute to the greater vulnerability faced by youth relative to other age groups [5]. One approach to reducing these barriers is the provision of mental health treatment via telehealth or remote platforms. The purpose of the assessment reported here was to test the preliminary effectiveness of remote intensive outpatient programming (IOP) in treating depression in youth with high acuity mental health diagnoses.

### Treating High Acuity Youth: IOP

IOP is an alternative model to inpatient care for youth with needs greater than those that can be served in traditional outpatient services. Youth attending IOP spend upwards of 15 hours per week in individual, family, and group treatment sessions with a multidisciplinary team of professionals, including mental health clinicians (psychiatrists, psychologists, counselors, and social workers), medical professionals (nurses and dietitians), and specialists trained in a variety of therapies such as art and recreational therapy [6]. Recent research suggests that the IOP model can effectively reduce crisis events, treat symptoms, improve overall functioning, and reduce readmissions to inpatient hospitals [6,7]. The IOP model can be particularly beneficial for youth and young adults because it allows them to continue to attend school or work, maintain developmentally important social relationships, and avoid out-of-home placement, an experience that can exacerbate unipolar depression regardless of placement type (acute or residential care) [8].

Although the IOP model can be an effective alternative to inpatient care for youth, place-based IOP locations are still limited to clients who live close enough to travel to the clinical setting several days per week. This can be particularly problematic for adolescents who depend on adults to drive them or provide the resources to take public transportation (bus or train fare) to the IOP location, assuming that public transportation is available. Furthermore, even shorter distances coupled with the expected frequency of attendance, can become burdensome on parents or caregivers and have negative impacts on the ability of parents to work and care for other children. Thus, adherence to IOP may be compromised.

Such geographic limitations restrict both the availability of services to youth living far from treatment settings as well as the degree to which treatment can be targeted to particular developmental and clinical needs, given that treatment groups by necessity reflect the conditions of the majority of the population in the particular geographic location. For example, a place-based IOP may not have enough similarly presenting clients to create a treatment group specifically for younger adolescents seeking treatment for eating disorders who identify as nongender conforming if most of the clients in that geographic area are older adolescents seeking treatment for anxiety who identify as cisgender.

### Remote IOP

Remote IOPs are intensive outpatient programs that conduct 100% of their services over Health Insurance Portability Accountability Act (HIPAA)–compliant video software, with patients and families typically attending sessions from home or school. Similar to place-based IOPs, clients attend 3-4 hours of treatment per day, 3-5 days per week. The primary benefit of remote IOP is the removal of geographic barriers inherent to place-based programs, which increases access to care for adolescents and young adults as well as allowing for the provision of more appropriate treatments specific to individual client needs. Because remote IOP can draw clients from an unlimited distance, it affords the opportunity to provide developmentally targeted treatment for youth at various points on the adolescent or young adult spectrum and specialized treatment for those whose demographic or clinical characteristics are unique. Due to its remote modality, populating a treatment group to the ideal therapeutic size is not dependent on the number of clients at the same stage of development with similar diagnoses, symptoms, and identities living in the same geographic area.

Previous research suggests that remote and internet-based interventions are effective in treating a variety of clinical diagnoses, with effect sizes equivalent to those reported for place-based or traditional face-to-face therapy [9]. More specifically, a systematic review of eTherapy concluded that web-based modalities are effective in treating depression in children and adolescents [10].

### Aims and Clinical Implications

The aim of this report is to present the findings of quality improvement (QI) analysis using data collected by program staff of a remote intensive outpatient program serving youth and young adults. The QI analysis presented here focused on changes in depression among clients diagnosed with depressive disorder to assess both overall treatment effectiveness and differences by subgroups, including those defined by gender and sexual orientation. The goal of the program’s ongoing QI efforts is to use outcomes data to inform clinical and programmatic decisions that will enable the program to better serve the needs of all program youth.

## Methods

### Overview

Outcomes data are regularly collected as a part of the program’s ongoing QI procedures and are reported both monthly and quarterly to their payors and providers. The data are also used to inform program and clinical changes to enhance the delivery of effective services. Data collected as part of the QI process were used to perform a repeated-measures, longitudinal assessment of changes in depression as measured by the Patient Health Questionnaire Modified for Adolescents (PHQ-A) at intake, discharge, and follow-up. Analyses tested changes in depression among all clients with depressive disorder and among subgroups. Threats to the validity of findings due to history and spontaneous remission were also explored.

### Ethics Approval

This project was reviewed and determined by the University of Pennsylvania Institutional Review Board to qualify as QI, indicating that these activities are not human subject research.

### Program Characteristics

The intensive outpatient program, *Charlie Health, Inc* (CH), whose data were used for the analyses is based in Montana but provides web-based mental health services to adolescents and young adults with high acuity mental health diagnoses nationwide. Clients are referred to CH primarily through direct relationships with other providers (eg, hospitals, care networks, and physicians). Clients also contact the company directly following web-based searches for intensive care.

The primary diagnoses served by the program include anxiety, depression, posttraumatic stress disorder, and bipolar disorder, with secondary diagnoses that include severe emotional disturbance, substance use disorders, and eating disorders. The program specializes in complex cases, including youth with severe trauma, neglect, juvenile justice, or foster care experience; gender and sexual minoritized youth; youth who reside in rural or native communities; and youth who have had multiple admissions to inpatient or hospital settings in the previous year.

A biopsychosocial assessment is completed for each client at intake after which they are placed in treatment tracks that include groups and therapeutic approaches identified as best practices for their developmental stage and specific diagnostic, identity, and behavioral health needs. Developmentally determined groups include those targeted to youth in early adolescence, middle adolescence, late adolescence, young adulthood, and emerging adulthood. Identity focused tracks and groups include those targeted to youth from minoritized gender and sexual orientation populations. Treatment modalities include variations of cognitive behavioral therapy, dialectical behavior therapy, and trauma-focused treatment. Client enrollment is ongoing and new clients may be placed into newly developed or already established groups. Additionally, because adjustments are made throughout the treatment process, clients may begin in one track but be reassigned to new groups based on their response to treatment.

Group therapies are provided in the following three 50-minute blocks, 3 days per week: evidence-based skill building interventions (ie, dialectical behavior therapy and cognitive behavioral therapy), general therapeutic processing, and experiential therapy (ie, art, music, and journaling). Individual and family therapy groups are also offered each week with masters-level, licensed clinicians. Parents and others involved in the client’s care are provided psychoeducation and mutual aid groups that deliver support and guidance in such topics as mindful communication and sibling support. Parents and caregivers are also provided weekly “IOP Roadmap(s),” which impart information on the skills their children are learning with tips on how to support the practice of those skills at home.

### Client Characteristics

Data for clients who were in treatment between June 2021 and October 2022 were reviewed for inclusion. Those clients with a diagnosis of depressive disorder who also passed engagement and completion criteria, defined as a minimum of 18 hours of treatment (7 sessions) and at least two weeks in care, were included. The cutoff at 18 hours was informed by research on neurological indicators of brain changes in response to cognitive and behavioral therapies [11-14]. Licensed clinicians make a diagnosis of MDD in accordance with Diagnostic and Statistical Manual of Mental Disorder, fifth edition criteria based on patient responses during the semistructured intake session and throughout the course of treatment.

Cases in which the client did not meet this minimum standard of engagement were not included. Clients who did not complete treatment (discharged due to lack of engagement or insurance denial, transfer to lower or higher level of care, left against clinical advice, etc) but were deemed as having passed the engagement criteria (at least 18 hours of IOP sessions) were included despite discharge status. Cases in which clients were absent from group for extended lengths of time (ie, 2-4 weeks), regardless of the reason (vacation, admission to higher level of care, disengaged, etc) were also included as long as they either met the minimum level of engagement prior to departure or, upon their return to treatment, attained a level of engagement that passed engagement criteria.

A majority of the clients included in the analysis lived in Washington State (n=303), Texas (n=163), Arizona (n=106), Montana (n=88), California (n=58), Idaho (n=55), and Pennsylvania (n=45). A smaller number were from Delaware (n=27), Illinois (n=21), Utah (n=18), New York (n=14), New Jersey (n=12), and Florida (n=10). Five or fewer clients lived in each of the following: Colorado, Wyoming, New Mexico, Ohio, Alabama, Georgia, Kentucky, Michigan, Nevada, Indiana, Louisiana, Minnesota, and North Carolina.

### Data Collection Procedures

Baseline measures of depression are collected by IOP staff after intake and prior to the first group session. Upon joining their first group session, clients are moved to a breakout room within the video platform where they meet with an outcomes coordinator who shares the link to the web-based intake survey with them. If a client is unable to open the link within the platform, they are sent the link via email or text messaging. The process, repeated for the discharge survey, is scheduled to occur at each client’s final group session.

If clients are unable to complete the survey prior to discharge, they are sent the link via email or text messaging and offered a US $25 Amazon gift card for their time. The decision to offer a gift card was made in recognition of the time and effort the program was asking of the clients in completing the survey on their own time, outside of program hours. The amount offered is within ethical standards that would not be considered to be unduly influential [15-17]. It would, however, be enough to increase the likelihood that clients would see completion of the survey to be worthy of their time and attention [16,17].

IOP staff download the completed survey responses at the end of each month for internal reports and to share with the analysis team. The data are deidentified by IOP staff prior to uploading the file to a secure folder shared with the analysis team by removing names and assigning unique ID numbers.

### Measures

Demographic characteristics are collected from respondents, including age, gender identity, and sexual orientation. Treatment characteristics are collected from administrative records and include diagnoses, intake and discharge dates, number of weeks in treatment, total number of sessions attended, and discharge status (ie, completed treatment, administrative discharge, transferred to a lower or higher level of care, or left against clinical advice).

### Framework of PHQ-A

The PHQ-A is used to assess depression prior to and after treatment. PHQ-A is a self-report measure that uses a 9-item depression severity scale asking how bothered respondents have been in the past 2 weeks by symptoms such as “feeling down, depressed, irritable, or hopeless” and having “thoughts that you would be better off dead, or of hurting yourself in some way.” Response options range from 0 (not at all) to 3 (nearly every day). The PHQ-A has been found to have good diagnostic validity [18], and the Patient Health Questionnaire (PHQ-9), upon which the adolescent modified scale is based, was found to have good criterion validity [19]. Reliability for the clients included in analyses was very good, with a Cronbach α of 0.89.

### Data Analysis Strategy

Descriptive statistics were used to summarize the client group as a whole. Means, SDs, minimums, and maximums were calculated for the following continuous variables: age, PHQ-A scores at intake, number of weeks, and treatment sessions attended. Frequencies were computed for the following categorical variables: comorbidity, symptom severity at intake, gender identity, sexual orientation, and discharge status.

### Program Effectiveness

To test the overall effectiveness of the remote intensive outpatient program to reduce depression, overall PHQ-A scores at intake and discharge were used as repeated measures and to calculate difference scores. Symptom severity scores based on overall PHQ-A scores at intake and discharge were used to identify movement across the clinical threshold associated with MDD. These 2 metrics, difference scores and clinical thresholds, are considered indices of clinically meaningful change as defined and studied by Wolpert et al [20].

Changes in depression scores between intake and discharge were analyzed employing a repeated-measures *t* test. Changes in symptom severity were used to identify whether clients crossed the clinical threshold associated with MDD symptoms [19,21,22]. The threshold was identified as a reduction from moderate, moderately severe, or severe symptom categories at intake to no or mild symptom categories at discharge [21]. McNemar chi-square test of independence was conducted to analyze categorical changes in symptom severity between intake and discharge.

### Subgroup Comparisons

One-way ANOVA was used to compare differences in treatment effects among self-reported gender and sexual orientation groups. Difference scores were calculated for use as the dependent variable by subtracting PHQ-A intake scores from PHQ-A discharge scores so that treatment gains would be demonstrated as negative scores, reflecting a reduction in depression.

Because adolescence and young adulthood encompass several developmental periods [23,24], differences in treatment effects by age were also explored. Age was collapsed into the following 3 groups that reflected different neurological and physiological stages of development: early adolescence (ages 11-14 years), associated with the onset of puberty [25]; middle adolescence (ages 15-18 years), associated with peak neurotransmitter activity in the limbic system [24]; and late adolescence or early adulthood (ages 19-25 years), associated with increased myelination in the self-regulatory areas of the prefrontal cortex [23,24]. The new *AgeGroup* variable was then included as the independent variable in an ANOVA with difference scores as the dependent variable.

### Threats to Validity

To test for possible effects of history, particularly those from seasonal differences, that could explain changes in depression between intake and discharge, an ANOVA was conducted that compared mean change scores by season of intake. To separate treatment experience into seasons, clients’ month of intake were used to assign them to 1 of 4 seasons as defined by the National Geographic Society [26]: Winter (December, January, and February), Spring (March, April, and May), Summer (June, July, and August), or Fall (September, October, and November). To test for spontaneous remission, 2 repeated-measures *t* tests were performed comparing depression scores at intake versus discharge and at discharge versus 3 months post discharge.

## Results

A total of 1062 cases met inclusion criteria and were included in analyses. The average treatment engagement lasted 10 (SD 4.12) weeks with a mean of 27 (SD 12.03) sessions. The average age of the clients was 16.2 (SD 3.3) years with a range of 11-25 years. As shown in Table 1, almost half (44%) of the clients identified as female, 23% identified outside of the male or female binary, and 20% identified as transgender people. A majority of the clients (60%) identified as a member of the lesbian, gay, bisexual, transgender, queer (LGBTQ+) community, with 30% classifying as heterosexual.

Based on inclusion criteria, all clients had a diagnosis of depression and as a group entered treatment with an average depression score of 14.90 (SD 7.13). Three quarters of the clients (73%) entered treatment experiencing moderate to severe symptoms, 22% entered with minimal to mild symptoms, and 2% reported no symptoms (see Table 2). A total of 60% of the clients also had a co-occurring diagnosis of anxiety, 5% had a co-occurring diagnosis of trauma-related stress, and 1% were also diagnosed with one of the following: cluster B personality disorder; alcohol use disorder; attention-deficit/hyperactivity disorder; bipolar disorder; cannabis disorder; disruptive, impulse, or conduct disorder; an eating disorder; or gender dysphoria.

**Table 1:** Client characteristics at intake.

			Participants, n (%)
**Gender identity**			
	Female	473 (44.5)	
	Gender-fluid	69 (6.5)	
	Gender-neutral	15 (1.4)	
	Gender-questioning	25 (2.4)	
	Genderqueer	15 (1.4)	
	Male	261 (24.6)	
	Nonconforming	19 (1.8)	
	Nonbinary	104 (9.8)	
	Missing	81 (7.6)	
**Sexual Orientation**			
	Asexual or graysexuality	49 (4.6)	
	Bisexual	231 (21.8)	
	Gay	34 (3.2)	
	Heterosexual or straight	319 (30)	
	Lesbian	55 (5.2)	
	Pansexual	164 (15.4)	
	Queer	58 (5.5)	
	Questioning	53 (5)	
	Missing	99 (9.3)	

**Table 2:** Severity of depression symptoms at intake.

	Participants, n (%)
None to minimal	103 (9.7)
Mild	151 (14.2)
Moderate	212 (20.0)
Moderately severe	255 (24.0)
Severe	309 (29.1)
Missing	32 (3.0)

### Program Effectiveness

As summarized in Table 3, depression scores were, on average, 6 points lower at discharge than at intake (*t*_968_=–24.68, *P*<.001). This difference equates to a large effect of treatment on depression (Cohen *d*=–0.79) and supports the effectiveness of the remote IOP to significantly reduce depression between intake and discharge.

**Table 3:** Analysis of differences in depression between intake and discharge.

			Mean (SD)		SE mean		Mean difference		*t* test (*df*)		*P* value (2-tailed)		Cohen *d*
**Depression score**							–6.06		–24.68 (967)		<.001		–0.79
	Intake	15.01 (7.11)		0.23									
	Discharge	8.95 (6.60)		0.21									

### Clinical Threshold

Table 4 summarizes the McNemar chi-square test of independence. A significant number of clients (*P*<.001) crossed the clinical threshold for MDD from intake to discharge (388/732, 53%), meaning that they decreased in symptom severity from moderate, moderately severe, or severe symptoms at intake to no or mild symptoms at discharge.

**Table 4:** Crossing the clinical threshold using symptom severity at intake versus discharge.^a^

			Discharge, n				Total, n
			None to mild	Moderate to severe			
**Intake**							
	None to mild	184		48	232		
	Moderate to severe	388		348	736		
Total			572	396		968	

^a^McNemar ^2^ (N=968): *P*<.001.

### Subgroup Comparisons

There were no significant differences found in analyses of change scores among subgroups of gender (*F*_7,886_=1.20; *P*=.30), sexual orientation (*F*_7,872_=0.47; *P*=.86), or age (*F*_2,958_=0.47; *P*=.63). This suggests treatment effects were equivalent across subgroups.

### Threats to Validity

Change scores were significantly different among clients by the season in which they started CH (*F*_3,964_=5.64, *P*<.001). Post season-by-season comparisons suggest that the significant difference was due to the smaller change in PHQ scores among those who started CH in the summer compared to those who started in the spring. Additional posthoc month-by-month comparisons found that the August 2021 PHQ change score was significantly and substantially smaller than over half of the 15 other intake months, including October 2021, January 2022, February 2022, March 2022, April 2022, May 2022, June 2022, and July 2021 (see Table 5 for additional detail). The only other significant difference among the 16 intake months was between December 2021 and May 2022. Given the substantially lower average change score for clients who began CH in August 2021 and the lack of difference among the other summer months (June 2021, July 2021, June 2022, July 2022, and August 2022) or between these other summer months and any spring months, the difference between summer and spring may be interpreted as a reflection of the significantly smaller change in depression that occurred in this single summer month (August 2021).

**Table 5:** One-way ANOVA of change scores between intake and discharge.

	Mean (SD)	Minimum	Maximum	Participants, n
Winter	–5.90 (8.47)	–27	23	183
Spring	–7.46^a^ (7.47)	–26	17	304
Summer	–5.06^a^ (7.18)	–27	24	354
Fall	–5.70 (7.55)	–23	13	127
June 2021	–5.38 (6.70)	–19	3	13
July 2021	–4.61 (8.00)	–27	9	28
August 2021	0.10 (7.19)	–10	24	30
September 2021	–4.39 (8.89)	–23	13	33
October 2021	–6.26^b^ (7.49)	–22	8	43
November 2021	–6.02 (6.63)	–19	7	49
December 2021	–2.86 (9.79)	–27	23	43
January 2022	–6.26^b^ (9.12)	–25	18	58
February 2022	–7.23^b^ (6.79)	–21	8	82
March 2022	–7.34^b^ (8.63)	–26	17	83
April 2022	–6.72^b^ (6.93)	–25	9	95
May 2022	–8.10^b^ (7.03)	–25	8	126
June 2022	–5.68^b^ (7.24)	–24	12	137
July 2022	–5.91^b^ (7.06)	–24	17	94
August 2022	–5.00 (5.90)	–17	10	52
September 2022	–7.50 (10.61)	–15	0	2

^a^Significantly different.

^b^Significantly different than August 2021.

### Spontaneous Remission

Table 6 summarizes the repeated-measures *t* tests analyzing differences in depression scores between intake and discharge and between discharge and follow-up among clients who had intake, discharge, and 3-month PHQ-A scores. The depression scores in this subgroup were significantly lower at discharge compared to intake by an average of 4.6 points (*t*_128_=–6.64, *P*<.001). Depression scores were not significantly different between discharge and 3-month follow-up. The significant reduction in depression found between intake and discharge but not between discharge and follow-up suggests that spontaneous remission, while still a possible factor, was not the only factor underlying reductions seen during the treatment period.

**Table 6:** Analysis of differences in depression between discharge and 3-month follow-up.

Depression score		Mean (SD)	SE mean	Mean difference		*t* test (df)		*P* value (2-tailed)	
**Intake vs discharge**					–4.61		–6.64 (128)		<.001
	Intake	14.66 (7.32)	0.65						
	Discharge	10.05 (6.50)	0.57						
**Discharge vs 3-month follow-up**					–0.09		–0.16 (128)		.88
	Discharge	10.05 (6.50)	0.57						
	3-month follow-up	9.95 (6.86)	0.60						

## Discussion

### Overview

The aim of the QI analysis undertaken here was to assess the effectiveness of a remote intensive outpatient program to treat depression in adolescents and young adults. Program effectiveness was supported such that depression was lower at discharge compared to intake as tested by significant changes in both PHQ-A scores and symptom severity. This aligns with and provides additional support for previous research suggesting that mental health needs can be effectively met using remote treatment models [9,10] Furthermore, these analyses provide support for the use of remote treatment with more acute (ie, IOP) clients coming to treatment with more complex mental health needs.

Subgroup comparisons provide support for remote IOP as a treatment option that is equally effective with adolescents and young adults who identify across gender and sexual orientation populations. This is important given that marginalized populations, including those who do not identify in the gender binary and those who select as members of LGBTQ+ communities, are at higher risk of mental health disorders due to experiences of marginalization [27,28] and encounter even greater barriers to appropriate mental health services than nonmarginalized groups [28-30]. Subgroup analysis also provides support for the use of the remote intensive outpatient program model with the range of developmentally unique groups commonly characterized under the umbrella term “adolescents and young adults” or “youth.” Analyses found treatment effects to be equivalent across age groups ranging from early adolescence (11-13 years of age) to young adulthood (19-25 years of age).

The equivalent treatment effects determined across subgroups are likely the result of both program factors that are common across all clients as well as those that address the unique needs of individuals. All clients experience the same treatment platform (remote) and model (intensive outpatient program) that increase their access to intensive services while lowering burdens associated with travel time and cost. At the same time, individual treatment plans are specific to the developmental, psychological, and psychosocial needs of each client, meaning that each client gets to experience treatment groups designed to address their unique intersection of needs related to diagnoses, symptoms, developmental period (age), and identity (gender and sexual orientation).

### History Effects

One threat to the validity of findings is history or exposure to factors outside of the IOP treatment process that could have reduced depression between intake and discharge [31]. These external events or experiences are only considered threats when all clients are exposed to them and, for the purposes of this analysis, are those that reduce depression. One common history effect for which there is evidence of an impact on depression and to which all clients would be exposed is related to seasonal variation, for instance weather differences or differences in longer versus shorter periods of daylight [32,33]. Because client treatment episodes were dispersed across 4 seasons, we were able to compare average reductions in scores by season. While findings indicated a significant difference between spring and summer in the average change in depression, further analysis suggested that a single summer month (August 2021) may have been the primary driver of the difference. Given that the average change in depression for clients who started in August 2021 occurred in a single summer month across 2 summers, the difference may be better explained by selection bias than by seasonal effects. Additionally, history effects are of concern when they mimic treatment effects, which is not the case here as whatever led to the difference in scores in August 2021 led to a *smaller* average change in depression rather than an increase as we would expect with history effects that mimic treatment effects. As such, it does not appear that seasonal effects explain the significant decrease in depression between intake and discharge.

### Spontaneous Remission

In a clinical setting, spontaneous remission is the tendency for symptoms, regardless of severity, to improve over time independent of treatment [34]. Although only a small subgroup of clients (n=129) had follow-up scores with which to compare changes during treatment to those after treatment, results indicated that the significant reduction between intake and discharge plateaued after discharge, remaining stable between discharge and 3-month follow-up. Scores did not continue to decrease, nor did they increase. It is therefore plausible that spontaneous remission was not solely responsible for the reductions in depression during the treatment period, that the leveling off of reductions between discharge and 3-month follow-up was more a factor of the end of active treatment engagement. This explanation is particularly likely given that the timing between intake and discharge varied across clients, suggesting that, if spontaneous remission was exclusively responsible for improvements in depression, remission would also have had to occur on the same varied timeline.

### Implications

Since the COVID-19 pandemic, telemental health care treatment options have become far more acceptable and viable. Although they do not alleviate all disparities, there are certain disparities that they can reduce. The remote platform decreases younger clients’ dependency on others, such as parents for transport or the availability of public transportation for their attendance.

Providing treatment remotely in the manner described here may have also attracted and helped engage LGBTQ+ youth. Previous research has found that LGBTQ+ youth are more likely than heterosexual cisgender youth to participate in web-based treatment [35] and often use web-based LGBTQ+ communities to explore their identities and connect with other LGBTQ+ people [36]. The comfort with web-based platforms and a treatment approach that enabled LGBTQ+ youth to connect with similarly identifying peers across the country in a way that is not always feasible in a given geographic location may help to explain the high rates of LGBTQ+ clients in the program.

Such services may also be easier to establish parent involvement due to decreased time commitment required without the travel to a physical location. Additionally, such involvement and the ability to pull from a wide geographical range makes it feasible for programs to hold targeted parent groups, such as the LGBTQ+ parent group offered by this program. These factors may have contributed to the effectiveness of this service for LGBTQ+ youth.

This treatment modality can also serve a wide range of ages without necessity for establishing separate facilities or specialized units for youth and young adults that require drawing on a broad geographical area. Consequently, it is far more feasible to develop groups remotely with youth who have similar developmental and demographic needs, as again these unique groups are not constrained by geographical location which by its very nature may not have the needed diversity. Given the comfort and technical savvy common in youth and young adults, this treatment modality has the potential to not only be a comfortable treatment alternative but one that is more attractive because it is tech-heavy.

### Strengths and Limitations

A primary strength of this analysis is the use of outcomes data collected as part of program administration by program staff, which increases the likelihood that findings reflect the actual remote IOP process uninfluenced by the presence of research staff and additional resources frequently infused into organizations during the research process. The presence of research staff during the investigation of controlled interventions has implications for fidelity and the ability of an intervention to continue to engender the same treatment effects once organizations are left to implement interventions on their own [37]. The use of program outcome data as was done here, however, removes issues related to changes in intervention delivery at termination of the research process and the possible issues of fidelity that accompany them, and increases the likelihood that similar treatment effects will continue post evaluation given that the same treatment processes and providers are ongoing.

The gold standard in mental health treatment is personalized treatment to address each client’s unique intersection of mental health and developmental and psychosocial needs. This makes good mental health treatment inherently heterogeneous. The value that QI analysis brings to the field is the degree to which outcomes reflect what is happening in a real-world treatment setting. QI cannot have the precision of a randomized controlled trial as delivered by a research team, because it is based on clinical and administrative data, but it nonetheless extends the knowledge base.

Another strength of this analysis is the use of 2 metrics to assess clinically meaningful change [20]. One of the challenges of clinical research is the identification of metrics that assess changes that are not just meaningful in research and hypothesis testing but are also important to clients’ experiences in their day-to-day lives [38]. Both metrics were based on the PHQ-A, which assesses changes in self-concept and emotions, which are more conceptual, as well as changes in behavior and experiences that connect more directly to daily functioning, such as sleeping, eating, the ability to concentrate, and having interest or taking pleasure in doing things, all of which are directly consequential to day-to-day living. It could also be argued that such changes are subjectively important and valued by the client, an essential goal in clinical research that can be lost when measures are only of theoretical constructs.

A primary limitation of this study is the lack of a comparison group with which to compare those in treatment to assure that changes over time were not due to factors other than or outside of the treatment experience. To account for this limitation, the following 2 threats to validity of the findings were explored in analyses: history effects and spontaneous remission.

### Conclusion

These analyses help to fill the gap in the literature regarding the use of remote mental health services (telehealth) to treat adolescents and young adults with more complex mental health needs by providing evidence for the ability of remote IOP to reduce depression in high acuity youth without hospitalization and with treatment effects that remained stable for up to 3 months post discharge. The metrics used to assess treatment effects reflect both changes in clinical diagnoses as well as changes in real-world symptoms, including changes in sleeping and eating patterns, the ability to concentrate, and having interest or taking pleasure in everyday activities, all of which have direct implications for the physical and mental well-being of clients in everyday life.

This study also provides evidence for the ability of remote intensive outpatient program to effectively treat those youth who identify as members of marginalized gender and sexual orientation populations. The opportunity to work outside of the limitations of place and local demographics allows clinicians to create specialized treatment groups sensitive to identity-specific needs and, given the disproportionately high percentage of clients who identified as a member of an LGBTQ+ community, may provide a safer space to self-disclose than those based in local facilities. The remote intensive outpatient program model as delivered here could move the field forward in providing more opportunities and safer spaces for youth from LGBTQ+ communities to receive identity-sensitive treatment and in doing so address the disparities that lead to the poorer outcomes and greater treatment barriers experienced by LGBTQ+ youth compared to their cisgender, heterosexual peers.

Finally, providing evidence for the effectiveness of remote IOP engenders support for another level within the spectrum of care that allows patients whose needs go beyond that which can be addressed in regular outpatient settings to receive treatment in their homes rather than inpatient treatment settings or hospitalization. This in turn enables youth to receive a higher level of intensive treatment without removing them from their everyday supports and the healthy, normative activities that are developmentally essential during adolescence and young adulthood.

## Abbreviations

CH: Charlie Health, Inc
IOP: intensive outpatient programming
LGBTQ+: lesbian, gay, bisexual, transgender, queer
MDD: major depressive disorder
PHQ: Patient Health Questionnaire
PHQ-A: Patient Health Questionnaire Modified for Adolescents
QI: quality improvement

## Data Availability

The data used for this report were collected by program staff as part of ongoing quality improvement efforts and are therefore not available for public use.

## References

[1] GBD results. Institute for Health Metrics and Evaluation.

[2] (2019). 2019 mental health client-level data (MH-CLD) annual report. Substance Abuse and Mental Health Services Administratio.

[3] Cummings JR, Case BG, Ji X, Marcus SC (2016). Availability of youth services in U.S. mental health treatment facilities. Adm Policy Ment Health.

[4] National mental health services survey. Substance Abuse and Mental Health Services Administration.

[5] GBD 2019 Adolescent Mortality Collaborators (2021). Global, regional, and national mortality among young people aged 10-24 years, 1950-2019: a systematic analysis for the Global Burden of Disease Study 2019. Lancet.

[6] Leffler JM, D'Angelo EJ (2020). Implementing evidence-based treatments for youth in acute and intensive treatment settings. J Cogn Psychother.

[7] Costa M, Plant RW, Feyerharm R, Ringer L, Florence AC, Davidson L (2020). Intensive outpatient treatment (IOP) of behavioral health (BH) problems: engagement factors predicting subsequent service utilization. Psychiatr Q.

[8] Dahl SK, Larsen JT, Petersen L, Ubbesen MB, Mortensen PB, Munk-Olsen T, Musliner KL (2017). Early adversity and risk for moderate to severe unipolar depressive disorder in adolescence and adulthood: a register-based study of 978,647 individuals. J Affect Disord.

[9] Barak A, Hen L, Boniel-Nissim M, Shapira N (2008). A comprehensive review and a meta-analysis of the effectiveness of internet-based psychotherapeutic interventions. J Technol Hum Serv.

[10] Stasiak K, Fleming T, Lucassen MF, Shepherd MJ, Whittaker R, Merry SN (2016). Computer-based and online therapy for depression and anxiety in children and adolescents. J Child Adolesc Psychopharmacol.

[11] Garrett A, Cohen JA, Zack S, Carrion V, Jo B, Blader J, Rodriguez A, Vanasse TJ, Reiss AL, Agras WS (2019). Longitudinal changes in brain function associated with symptom improvement in youth with PTSD. J Psychiatr Res.

[12] Nahman-Averbuch H, Schneider VJ, Chamberlin LA, Kroon Van Diest AM, Peugh JL, Lee GR, Radhakrishnan R, Hershey AD, King CD, Coghill RC, Powers SW (2020). Alterations in brain function after cognitive behavioral therapy for migraine in children and adolescents. Headache.

[13] Roberts H, Jacobs RH, Bessette KL, Crowell SE, Westlund-Schreiner M, Thomas L, Easter RE, Pocius SL, Dillahunt A, Frandsen S, Schubert B, Farstead B, Kerig P, Welsh RC, Jago D, Langenecker SA, Watkins ER (2021). Mechanisms of rumination change in adolescent depression (RuMeChange): study protocol for a randomised controlled trial of rumination-focused cognitive behavioural therapy to reduce ruminative habit and risk of depressive relapse in high-ruminating adolescents. BMC Psychiatry.

[14] Tang Y, Lu Q, Fan M, Yang Y, Posner MI (2012). Mechanisms of white matter changes induced by meditation. Proc Natl Acad Sci USA.

[15] Dvořáková K, Kishida M, Li J, Elavsky S, Broderick PC, Agrusti MR, Greenberg MT (2017). Promoting healthy transition to college through mindfulness training with first-year college students: pilot randomized controlled trial. J Am Coll Health.

[16] Henderson M, Wight D, Nixon C, Hart G (2010). Retaining young people in a longitudinal sexual health survey: a trial of strategies to maintain participation. BMC Med Res Methodol.

[17] Lankenau SE, Sanders B, Hathazi D, Bloom JJ (2010). Recruiting and retaining mobile young injection drug users in a longitudinal study. Subst Use Misuse.

[18] Johnson JG, Harris ES, Spitzer RL, Williams JB (2002). The patient health questionnaire for adolescents: validation of an instrument for the assessment of mental disorders among adolescent primary care patients. J Adolesc Health.

[19] Kroenke K, Spitzer RL, Williams JBW (2001). The PHQ-9: validity of a brief depression severity measure. J Gen Intern Med.

[20] Wolpert M, Görzig A, Deighton J, Fugard AJ, Newman R, Ford T (2015). Comparison of indices of clinically meaningful change in child and adolescent mental health services: difference scores, reliable change, crossing clinical thresholds and 'added value'--an exploration using parent rated scores on the SDQ. Child Adolesc Ment Health.

[21] Manea L, Gilbody S, McMillan D (2012). Optimal cut-off score for diagnosing depression with the patient health questionnaire (PHQ-9). Can Med Assoc J.

[22] Urtasun M, Daray FM, Teti GL, Coppolillo F, Herlax G, Saba G, Rubinstein A, Araya R, Irazola V (2019). Validation and calibration of the patient health questionnaire (PHQ-9) in Argentina. BMC Psychiatry.

[23] Luna B, Padmanabhan A, O'Hearn Kirsten (2010). What has fMRI told us about the development of cognitive control through adolescence?. Brain Cogn.

[24] Shulman EP, Harden KP, Chein JM, Steinberg L (2015). Sex differences in the developmental trajectories of impulse control and sensation-seeking from early adolescence to early adulthood. J Youth Adolesc.

[25] Graber JA, Nichols TR, Brooks-Gunn J (2010). Putting pubertal timing in developmental context: implications for prevention. Dev Psychobiol.

[26] Season. National Geographic Society.

[27] Hunter J, Butler C, Cooper K (2021). Gender minority stress in trans and gender diverse adolescents and young people. Clin Child Psychol Psychiatry.

[28] McCabe HA, Kinney MK (2019). LGBTQ+ individuals, health inequities, and policy implications. Creighton Law Rev.

[29] Clark KD, Capriotti MR, Obedin-Maliver J, Lunn MR, Lubensky ME, Flentje A (2019). Supporting sexual and gender minority health: research priorities from mental health professionals. J Gay Lesbian Ment Health.

[30] Haney JL (2021). Sexual orientation, social determinants of health, and unmet substance use treatment need: findings from a national survey. Subst Use Misuse.

[31] Jhangiani R, Chiang I, Cuttler C, Leighton D (2019). Research Methods in Psychology, 4th edition.

[32] Lukmanji A, Williams JV, Bulloch AG, Bhattarai A, Patten SB (2019). Seasonal variation in symptoms of depression: a Canadian population based study. J Affect Disord.

[33] Lukmanji A, Williams JV, Bulloch AG, Patten SB (2020). Seasonal variation in specific depressive symptoms: a population based study. J Affect Disord.

[34] Whiteford HA, Harris MG, McKeon G, Baxter A, Pennell C, Barendregt JJ, Wang J (2012). Estimating remission from untreated major depression: a systematic review and meta-analysis. Psychol. Med.

[35] Rosenthal S Breaking down barriers: young adult interest and use of telehealth for behavioral health services. World Health Organization.

[36] Fish JN, Williams ND, McInroy L, Paceley MS, Edsall RN, Devadas J, Henderson SB, Levine DS (2022). Q Chat Space: assessing the feasibility and acceptability of an internet-based support program for LGBTQ youth. Prev Sci.

[37] Altman DG (1995). Sustaining interventions in community systems: On the relationship between researchers and communities. Health Psychol.

[38] Kazdin AE (2006). Arbitrary metrics: implications for identifying evidence-based treatments. Am Psychol.

